# Overcoming cognitive set bias requires more than seeing an alternative strategy

**DOI:** 10.1038/s41598-022-06237-0

**Published:** 2022-02-09

**Authors:** Sarah M. Pope-Caldwell, David A. Washburn

**Affiliations:** 1 Department of Comparative Cultural Psychology, Max Planck Institute for Evolutionary Anthropology, Leipzig, Germany; 2 Language Research Center, Department of Psychology, Georgia State University, Atlanta, GA USA

**Keywords:** Psychology, Human behaviour

## Abstract

Determining when to switch from one strategy to another is at the heart of adaptive decision-making. Previous research shows that humans exhibit a ‘cognitive set’ bias, which occurs when a familiar strategy occludes—even much better—alternatives. Here we examined the mechanisms underlying cognitive set by investigating whether better solutions are visually overlooked, or fixated on but disregarded. We analyzed gaze data from 67 American undergraduates (91% female) while they completed the learned strategy-direct strategy (LS-DS) task, which measures their ability to switch from a learned strategy (LS) to a more efficient direct strategy (DS or shortcut). We found that, in the first trial block, participants fixated on the location of the shortcut more when it was available but most (89.6%) did not adopt it. Next, participants watched a video demonstrating either the DS (*N* = 34 *Informed* participants) or the familiar LS (N = 33 *Control*s). In post-video trials, *Informed* participants used the DS more than pre-video trials and compared to *Controls*. Notably, 29.4% of *Informed* participants continued to use the LS despite watching the DS video. We suggest that cognitive set in the LS-DS task does not stem from an inability to see the shortcut but rather a failure to try it.

## Introduction

Humans live in a range of diverse and dynamic environments. Adaptive decision-making hinges on cognitive flexibility, the ability to select between known strategies and innovated or acquired novel strategies to meet changing demands^1,2^. When a known solution stops working, switching to another is clearly beneficial. Even from a young age, humans are adept at switching when instructed to do so^3,4^ or after receiving feedback that a current strategy is no longer effective^5^. However, these *forced-switch* contexts are not the only time when changing tact is beneficial. In dynamic environments, the effectivity of a strategy is likely to change over time and although a familiar strategy continues to be useful, alternatives may eventually be better. Under *optional-switch* contexts, when a current strategy works but others are available, it can be difficult to know if, or when, to switch to an alternative.

Moreover, searching for an alternative strategy can be time-consuming or risky, and even if a viable alternative is found, the time invested in finding and learning a new strategy might easily outweigh the benefits of using it—especially in the short-term. Balancing the tradeoffs of exploiting a current strategy and exploring alternatives is a fundamental challenge that plagues diverse fields ranging from ecology^6,7^ to data science^8,9^. Humans minimize the cognitive resources spent deciding when to stay and when to switch strategies by relying on rules-of-thumb, or heuristics^10,11^. However, this ‘mechanized’ approach can lead us astray.

In optional-switch contexts, humans often exhibit a ‘cognitive set’ bias, which occurs when a familiar strategy occludes—even much better—alternatives^12,13^. For example, after learning to solve a set of ‘water jar’ math problems using a four-step method, Luchins^14^ found that thousands of participants, from a variety of age-groups and backgrounds, were blinded to a better one-step alternative. This finding has been widely replicated^15–19^ and extends beyond mathematics to other areas of cognition, including strategic reasoning^20–22^ design and engineering^23–25^, spatial navigation^14,26^, tool-use^27–29^, as well as insight^30,31^, lexical^14,32^, and sequential problem solving^12,13,33–35^. Cognitive set bias is pervasive, but its underlying mechanisms remain unclear.

One hypothesis is that cognitive set affects visual search, such that more time is spent looking at stimuli relevant to the familiar method than alternatives. In other words, once a strategy is adopted, alternatives are to some extent overlooked. For example, Bilalić et al.^20^ found that chess experts’ gaze remained primarily focused on pieces involved in a familiar checkmate pattern, the smothered mate, rather than a better alternative—despite reporting that they were looking for other solutions. Similarly, Knoblich et al.^36^ found that when presented with ‘matchstick’ arithmetic problems, where moving a single line is necessary to balance an equation (e.g. IV = III + III, solution: VI = III + III), participants who struggled to solve problems that required the disassembly of operators (e.g. using + to create =) looked at the key operators no more than would be expected by chance. These studies support the idea that cognitive set is accompanied by visual or attentional bias towards familiar solutions. However, efforts to reduce cognitive set by increasing the saliency of alternatives, show limited success^14,18,37^.

Another hypothesis is that cognitive set arises from an unwillingness to search for alternatives. This might be due to an assumption that the current strategy is the only, or best, available strategy, and therefore, the cost of searching for another is prohibitive. In other words, cognitive set arises from a prediction error stemming from similar prior experience or assumptions about the current situation. Luchins^14^ found that participants who were told “Don’t be blind” prior to being given the water jar problems used the shorter method more than controls, who persisted with the familiar long solution. He noted that cognitive set (referred to as “Einstellung”) may have arisen because “[Participants] were not accustomed to being taught one method and [then] expected to seek for, or use other methods.” For example, after Luchins showed participants the shorter method, some replied, "You did not teach us that method," or "You should have shown the other way, too, if you wanted us to use it," (p. 90). Likewise, Knoblich et al.^36^ suggested that cognitive set in matchstick problems likely stems from participants’ prior experience with operators as “constant elements” (p. 1008).

In the current study, we investigated whether cognitive set arises because better solutions are visually overlooked, or fixated on but disregarded. Participants completed the Learned Strategy-Direct Strategy (LS-DS) task, a nonverbal, nonmathematical adaptation of Luchins’^14^ water jar task, which measures their propensity to switch from a learned strategy (LS) to a more efficient direct strategy (DS or shortcut) when it becomes available. The LS-DS task has been shown to elicit high rates of cognitive set in American participants, but interestingly, several non-human primate species seem relatively unaffected^12,13,34,35^. Here, we tracked participants’ gaze while they completed the LS-DS task to test the hypothesis that cognitive set is driven by a visual/attentional bias occluding the shortcut. Additionally, in the second half of the experiment, we measured shortcut-use following a video demonstration of the DS, compared to controls who saw a video of the LS, to assess whether cognitive set would be broken if we removed the costs of searching for a new strategy.

## Methods

### Participants

Data were collected from 72 participants, recruited from the pool of undergraduate students at Georgia State University through the SONA Experiment Management System. Originally, the minimum sample-size was determined to be 60 participants, based on a frequentist power analysis (power = 0.85, cohen’s f = 0.25); although we later decided to use Bayesian modeling, this sample-size yielded satisfactory model fits throughout the analyses. Participants were pseudo-randomly assigned to the *Control* and *Informed* conditions, with the requirement that an equal number of males and females were assigned to each. Five participants were excluded from all analyses as a result of either technical malfunctions (*N* = 3), opting out (*N* = 1), or not passing training (*N* = 1). The final data set includes 67 participants [mean 20.2 (SD 4.0) years, 91% female]. Previous research using the LS-DS task has found no evidence of sex-differences in participants’ strategy-use^13,34,35^; but it should be noted that the current sample is heavily biased towards female participants and is therefore not demographically representative of the underlying population. For three participants, no eye tracking data were available due to system error; however, their response data were included in non-gaze analyses.

### Procedure

The study was approved by the Georgia State University Institutional Review Board for human subjects and all methods were performed in accordance with the applicable institutional, national, and international guidelines for ethical human research. Informed consent was obtained prior to testing. Testing occurred on Georgia State University campus in a private room with dimmed lights. Participants sat approximately 60 cm from a 19inch monitor (1280 × 1040 Native Resolution; 33.8 × 27.1 cm display size; 1915L Desktop Touchmonitor, Elo Touch Solutions). Responses were collected via mouse clicks to minimize movement. Gaze was captured by the Eye Tribe Tracker (The Eye Tribe), using 16-point gaze calibration. The experiment was conducted in OpenSesame Experiment Builder (version 3.1.1; Mathot et al.^38^), with the PyGaze plugin (version 0.6.0a16; default settings). Prior to testing, participants were informed of the audio and visual cues for correct and incorrect responses and told that they would need to “select the shapes to figure out the right answer.” Incorrect cues appeared immediately following any incorrect selection during a participants’ response, followed by a 3 s delay and a new trial. Correct cues were only elicited after participants had correctly completed the trial, (i.e. correct intermediate steps were not indicated, except by the lack of incorrect feedback). To start each trial, participants looked at the fixation cross (within a 1.5° threshold) while pressing the SPACE bar; this was enforced by the PyGaze drift-correct feature. If, at any point during the task, the eye tracker was unable to detect fixation after several attempts, the experiment was paused and gaze recalibrated. No further instructions were provided and the experimenter remained in an adjacent room (out of sight) unless recalibration was required.

### The LS-DS task

The LS-DS task began with three training levels, in which participants learned the three-step LS (Square1 → Square2 → Triangle) through trial-and-error. Throughout training and testing, the twenty-four possible configurations for the locations of Square1 (10 × 11 cm), Square2 (10 × 11 cm), and the Triangle (10 × 11 cm) were randomly presented. Participants progressed through training by achieving ≥ 80% accuracy, assessed every 8 trials. Training 1, 2, and 3 required a median of 8 (range 8–168), 8 (range 8–24), and 8 (range 8–32) trials, respectively.

After training, participants completed the first 48 experimental trials (Supplementary Fig. S1) while gaze was recorded (sample rate = 30 Hz). Experimental trials were presented in random order, and consisted of 24 baseline (BASE) trials, wherein the Triangle was not revealed until after Square 1 and Square 2 had been correctly selected, and 24 test (PROBE) trials, wherein the Triangle appeared alongside the Square 1 → Square 2 demonstration and remained visible throughout participants’ response. Crucially, on PROBE trials, participants could either continue to use the full LS (Square1 → Square2 → Triangle) sequence or they could skip Square1 → Square2 and simply select the Triangle (DS or shortcut). Thus, the LS-DS task assessed participants’ propensity to forego their learned response, the LS, in order to adopt the more efficient shortcut, the DS, when it was available (i.e. PROBE trials). Note that throughout training and testing, participants received immediate negative feedback after choosing any incorrect Square; however, selection of the Triangle–whenever it was available–always elicited the correct feedback cues. See Pope et al.^12^ for detailed task description.

After the first 48 (PRE) experimental trials, participants were given a questionnaire asking them to describe the role of various task components (e.g. the red Squares, Triangle, fixation cross) and their thoughts regarding the goal of the task in general. Once the participants completed the questionnaire (~ 5 to 10 min), they were shown a brief video twice, demonstrating either the DS [*Informed* group, N = 34, mean 20.8 (SD 5.1) age in years, 91% female] or the LS [*Control* group, *N* = 33, mean 19.6 (SD 2.3) years, 91% female] being performed in four consecutive PROBE trials. After the video, participants completed an additional 48 (POST) trials, again consisting of 24 BASE and 24 PROBE trials, randomly presented, followed by another, identical questionnaire. Thus, each participant completed the three training levels, 48 PRE trials, a PRE questionnaire, 48 POST trials, and a POST questionnaire, with eye-tracking recorded during all PRE and POST trials.

### Data processing

Gaze data were separated into mutually exclusive trial-parts: demo1, when the location of Square1 was shown; demo2, when the location of Square2 was shown; response1, the time until the participant’s first selection, response2, the time until the participant’s second selection; response3, the time until the participant’s third selection. For example, response1 consisted of all gaze data collected from the onset of the response screen until the first response. Fixations were determined based on the default Pygaze settings, which utilize the initial calibration procedure to detect fixations versus saccadic movements that exceed a participant-specific precision threshold (see Dalmaijer et al. ^39^). All non-fixations were excluded. Fixations were categorized into four regions of interest, corresponding to each quadrant of the screen: top left, top right, bottom left, and bottom right—excluding those that fell into the middle 30 pixels extending across the screen both vertically and horizontally, including the central fixation cross (Supplementary Fig. S2).

For all correct trials, we marked whether the LS (Square1 → Square2 → Triangle) or DS (Triangle) was used. However, on rare occasions, fewer than 1% of trials, a subset of participants (N = 11) selected Square 1, skipped Square 2, and then selected the Triangle—previously referred to as the “switch strategy” or SS^35^. Because Square1 → Triangle is a partial shortcut, we opted to group SS trials with DS trials for behavioral analyses. However, unlike typical DS trials, the first response is not the Triangle; thus, we excluded them from gaze and response time analyses performed on response1 data.

Questionnaires (both PRE and POST) were analyzed for indications that participants (1) noticed the Triangle appearing earlier in PROBE trials, or (2) ascribed more importance to the Triangle than other task components. For example, statements like “the triangle was sometimes a distraction” were categorized as noticing the early presence of the Triangle in PROBE trials. Responses noting that the Triangle was “how to progress to the next trial”, “how you knew you were correct”, or “the goal” were considered indications of ascribing importance to the Triangle. Participants received two scores for both PRE and POST questionnaires: noticed (yes) or not (no) and ascribed importance (yes) or not (no). The experimenter was blind to video condition during coding and a subset (21%) of the questionnaires were re-coded by another, condition-blind experimenter. A Spearman rank order correlation between the two observers indicated that scoring was reliable (*r*_*s*_ = 0.781, *p* < 0.001).

### Data analysis

All models were fit in *R version 4.0.3* using the *brms* (version 2.14.4^40,41^ interface with r*stan*^42^. Competing models were compared using the Widely Applicable Information Criterion (WAIC)^43^. Deidentified data are available here.

First, we assessed whether participants looked at the Triangle shortcut when it was available. Participants’ gaze data were used to determine if they fixated on the location of the Triangle (1) or not (0), prior to their first response. Using binary logistic regression models, we assessed whether Triangle fixations occurred more often in PROBE compared to BASE trials. Recall that the Triangle was not visible during response1 in BASE trials, so this comparison controls for random fixations on the Triangle’s location. Data were filtered to include only PRE trials in which the LS was used, to avoid video-condition or response strategy effects. Model 1.0 (Supplementary Table S1) included only the random effect of subject number. Model 1.1 also included the main effect of trial type (BASE or PROBE). Identical investigation of Square1, Square2, and Foil fixations are included in supplementary analyses (Supplementary Table S2).

Next, we investigated how shortcut-use was influenced by watching the video of either the DS (*Informed*) or the LS (*Control*). Using binary logistic regression models, we looked at whether participants’ use of the shortcut (1) vs the LS (0) differed between PRE and POST trial blocks, and as a function of their video condition (*Informed* vs *Control*). Trials with incorrect responses were excluded (PRE: mean 3.5, SD 3.7, max 18 trials; POST: mean 2.5, SD 2.5, max 10 trials). Model 2.0 (Supplementary Table S3) included only the random effect of subject number. Model 2.1 also included the main effects of block (PRE and POST) and video condition (*Informed* vs *Control*). Model 2.2 also included the interaction effect of block * video condition. Additionally, we ran a series of point-biserial correlations to investigate whether participants’ reports of noticing the Triangle (noticed) or ascribing importance to it (valued) correlated with shortcut use (Supplementary Table S4).

Next, we ran a set of Gamma regression models on overall trial times, to confirm that LS trials took longer than DS or SS trials. Responses shorter than 200 ms or longer than 3 times subjects’ total trial time standard deviation were excluded. Model 3.0 (Supplementary Table S5) included only the random effect of subject number on total trial time. Model 3.1 also included the main effect of strategy-used (LS, SS, or DS).

Finally, we explored whether switching between the LS and the DS resulted in switch costs, which are deficits in response time or accuracy that occur when switching from one strategy to another, compared to repeating the same strategy^44,45^. We fit a series of Gamma regression models, to see if the latency to first response was shorter in trials where participants repeated their previous strategy (DS preceded by DS, or LS preceded by LS) compared to trials where they switched strategies (DS preceded by LS, or LS preceded by DS). Responses shorter than 200 ms or longer than 3 times subjects’ response1 standard deviation were excluded. Only consecutive PROBE DS (*N* = 464) or BASE LS (*N* = 557) trials from participants that used the DS in more than 50% of trials during that trial block (*N*_*PRE*_ = 6, *N*_*Post*_ = 27) were analyzed. Model 4.0 (Supplementary Table S6) included only the random effect of subject number on response time. Model 4.1 also included the main effects of trial type (BASE or PROBE) and switch type (stay or switch). Model 4.2 also included the interaction effect of trial type * switch type.

## Results

In PRE trials, participants used the shortcut in a mean 9.56% (SD 25.39%) of correct PROBE trials. Only 7 out of 67 participants (10.45%; N_*Informed*_ = 4, N_*Control*_ = 3) used the shortcut in more than 50% of PROBE trials (Table S7); this is in line with previous findings^13,34,35^ and our expectations.

### Triangle fixations

**Figure 1:**
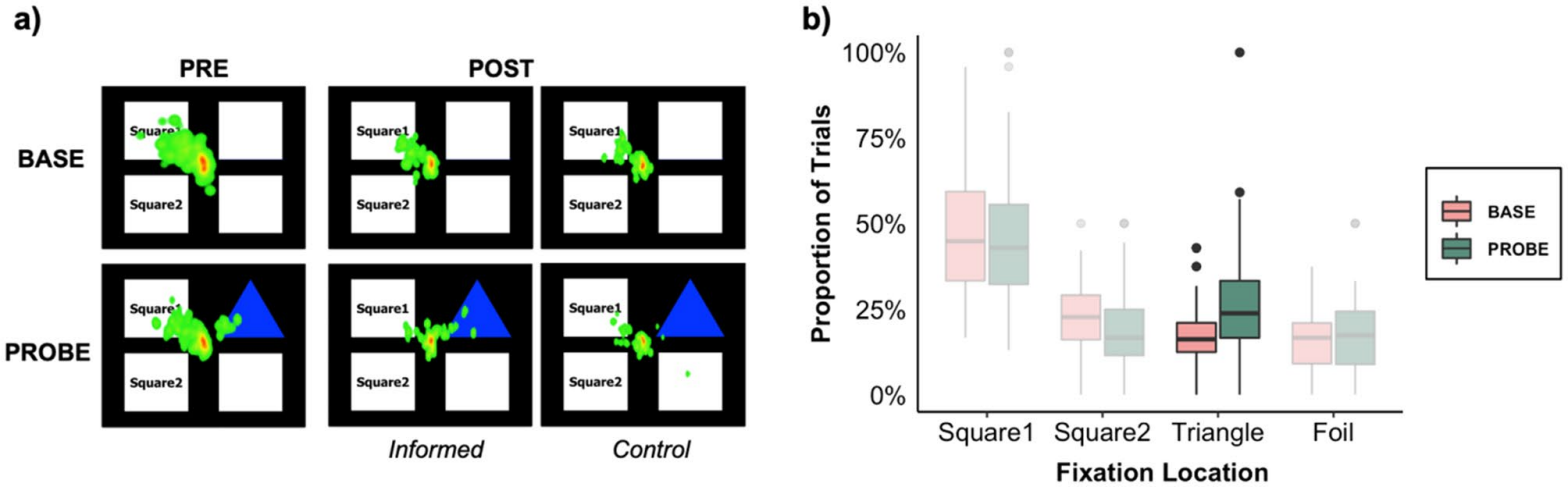
Gaze data during the LS-DS task. (**a**) All fixations, including those directed at the midline, that occurred prior to participants’ first response were compiled across all participants during the top left (Square1), bottom left (Square2), top right (Triangle) BASE and PROBE trial configurations, in PRE and POST trial blocks and (**b**) the proportion of PRE trials that participants fixated on each item, prior to using the LS, in BASE and PROBE trials.

First, we investigated whether participants continued to use the LS in PRE-PROBE trials because they did not see that the Triangle was already available. Model 1.1 had the lowest WAIC value (Supplementary Table S1). It found that, prior to using the LS, participants were more likely to fixate on the Triangle’s location in PROBE ((μ_PROBE_) = 0.53, CI = [0.34, 0.72]) compared to BASE trials (Fig. 1). This indicates that participants likely saw the Triangle in PROBE trials, but continued to use the LS. The converse was also true, Model S1 found that participants were slightly less likely to fixate on Square 1’s location in PROBE compared to BASE trials ((μ_PROBE_) = − 0.17, CI = [− 0.33, − 0.01]). There was no effect of trial type on fixations directed at Square 2 or Foil locations (Supplementary Table S2).

### Video information

**Figure 2:**
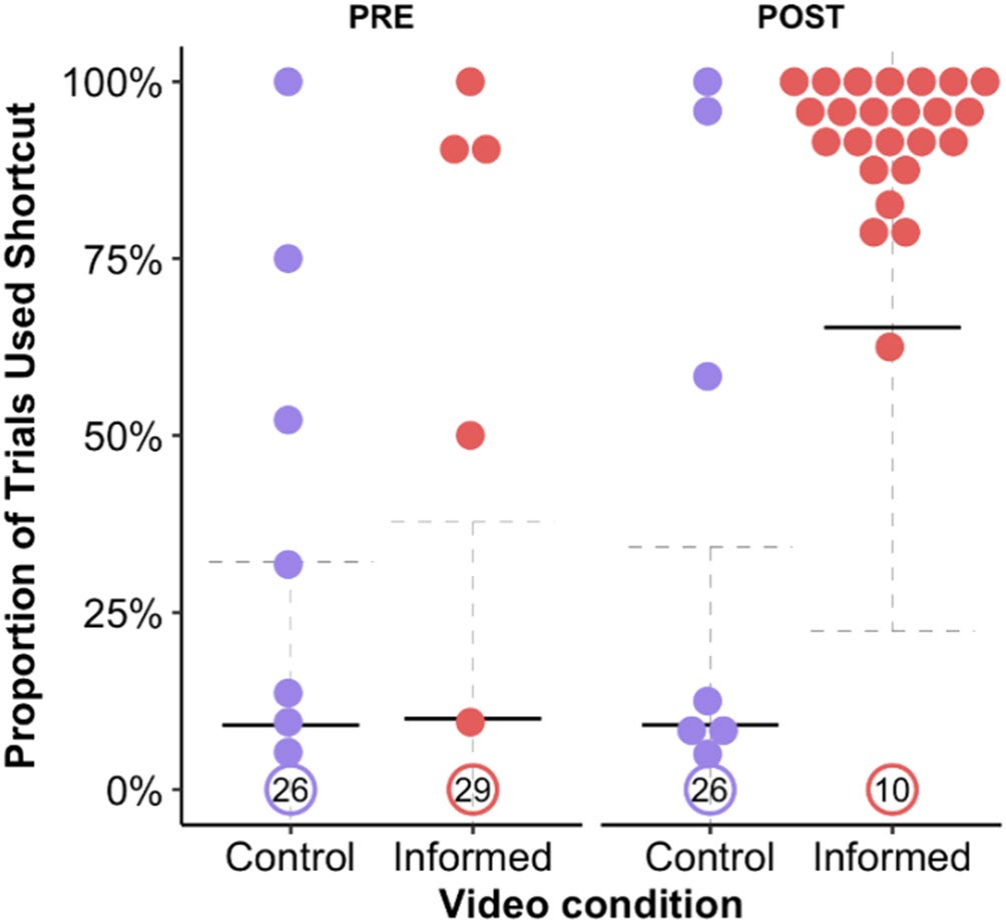
Shortcut-use during the LS-DS task. Mean proportion of trials that a shortcut (DS or SS) was used, for each subject in *Informed* and *Control* conditions, in PRE and POST trial blocks. Solid and dashed lines represent group means and standard deviations, respectively. Participants who used the shortcut in fewer than 5% of trials have been aggregated into counts at the bottom.

Next, we looked at whether watching the video demonstration of the shortcut increased participants’ ability to use it. In POST trials, *Control* and *Informed* participants used the shortcut in a mean 9.12% (SD 25.13%) and 65.25% (SD 42.88%) of correct PROBE trials, respectively. Only 3/33 (9.09%) participants that watched the video of the LS (*Control* group) used the shortcut in more than 50% of their correct trials. In stark contrast, 24/34 (70.59%) *Informed* participants used the shortcut in more than 50% of their correct POST trials (Table S7). Model 2.2 had the lowest WAIC value (Supplementary Table S3). It confirmed that, in POST trials, *Informed* participants were far more likely to use the DS ((μ_POST *Informed*_) = 6.06, CI = [5.23, 6.93]) than PRE *Control*, PRE *Informed* or POST *Control* participants (Fig. 2). However, note that 10/34 (29.41%) of *Informed* participants saw the video of the DS but still did not adopt it.

### Shortcut-use and self reports

We found no correlation between shortcut-use and noticing (*r*(67) = 0.17, *p* = 0.17) or valuing (*r*(67) = 0.19, *p* = 0.13) the Triangle in PRE trials. However, in POST trials, there was a small positive correlation between shortcut-use and noticing (*r*(67) = 0.30, *p* = 0.01) and especially for valuing (*r*(67) = 0.45, *p* < 0.001) the Triangle. See Tables 1 and S4.

**Table 1:** Participants’ self-reports of noticing the difference between BASE and PROBE trials (noticed), and of ascribing importance to the Triangle (valued).

	Noticed	Valued	Total
**PRE**			
Used DS ≥ 50%_trials_	5 (71.4%)	3 (42.9%)	7
Used DS < 50%_trials_	29 (48.3)	13 (21.7%)	60
**POST**			
*Control*			
Used DS ≥ 50%_trials_	1 (33.3%)	2 (66.7%)	3
Used DS < 50%_trials_	9 (30%)	9 (30%)	30
*Informed*			
Used DS ≥ 50%_trials_	14 (58.3%)	19 (79.2%)	24
Used DS < 50%_trials_	2 (20%)	3 (30%)	10

### Costs and benefits of shortcut-use

We confirmed that LS trial durations were much longer than DS ((μ_LS_) = − 0.82, CI = [− 0.90, − 0.74]) and SS trial durations ((μ_LS_) = − 0.38, CI = [− 0.65, − 0.11]; Supplementary Table S5); in other words, using either shortcut resulted in a faster trial. Finally, we looked at the switch costs associated with using the shortcut. Model 4.1 had the lowest WAIC values (Supplementary Table S6). It suggests, although tentatively, that switching between LS and DS strategies resulted in a slightly slower first response time ((μ_LS_) = 0.09, CI = [− 0.01, 0.20]) compared to trials in which participants repeated their previous strategy. However, these results should be interpreted cautiously.

## Discussion

The current study replicated previous reports of cognitive set in the LS-DS task^12,13,34,35^. Specifically, 89% of American adult participants persisted with an inefficient but familiar strategy—the LS, despite the availability of the more efficient shortcut—the DS. We tracked participants’ gaze while they completed the LS-DS task to test the hypothesis that cognitive set stems from visual bias towards familiar strategies, resulting in the shortcut being overlooked. However, this was not supported. Prior to using the LS, participants often fixated on the location of the Triangle, suggesting that they saw the shortcut but still did not use it. Next, we measured shortcut-use following a video demonstration of the DS, to look at whether cognitive set would be broken once participants learned that the DS was a viable alternative. These *Informed* participants were 24 × times as likely to use the shortcut as *Controls*, who were shown a video of the LS. However, for 10 *Informed* participants (29.4%), shortcut-use remained negligible, mean 0.85% (SD 1.80%) of correct POST trials.

Our finding that participants continued to use their familiar solution even after fixating on the Triangle, is contradictory to previous reports. Specifically, using chess and arithmetic paradigms, prior studies concluded that participants’ cognitive set arose because they were not looking for alternatives, as indicated by their gaze^20,36^. We suggest that in these paradigms, the alternative strategy was no more visually salient than the familiar approach, making it difficult to discern between participants’ not seeing the alternative versus not looking for it. However, in the LS-DS task the shortcut is very salient—the Triangle is the only icon on the screen during the response phase of PROBE trials (Fig. 1a). The current study found that, prior to using the LS, participants were 1.5 × more likely to fixate on the Triangle’s location during PROBE trials. Although fixations are only proxies for visual attention, and fixations on the Triangle might reasonably occur simply because of its saliency or role as the third response item when using the LS, our findings indicate that seeing the Triangle was not enough to prompt its use as a shortcut. Instead, we propose that cognitive set on the LS-DS task stems from a reluctance to explore alternative solutions.

Sampling alternatives can be time-consuming and risky. If a working strategy is already in place, these costs may not be outweighed by the mere *possibility* of a better solution. One way that humans mitigate the risks of decision-making under uncertainty, is by using problem-solving heuristics or rules-of-thumb that are based on previous experience with similar situations^10,11^ and are subject to individual differences^46^. Specifically, in problem spaces or environments which change often or are otherwise uncertain, alternative strategies may be sampled frequently^47,48^ or reliance on other sources of information, like socially-acquired strategies can increase^49^.

We suggest that in the LS-DS task, and likely many other instances of cognitive set, failure to use the alternative strategy stems from a prediction error that leads participants to overestimate the stability or predictability of the problem space—in other words, participants believe that the best strategy at the beginning of the game will continue to be the best strategy throughout the game*.* Indeed, Pope et al.^12^ found that telling participants “Don’t be afraid to try new things”, resulted in a substantial increase in shortcut use for American participants. Similarly, Luchins noted that higher rates of cognitive set were often found in children enrolled in remedial arithmetic, wherein rote practice was used extensively^14^. The current study recruited undergraduate students from a Western, Educated, Industrialized, Rich, Democratic (WEIRD)^50^, urban context, and tested them on university property. Perhaps it is not surprising that, even after watching the video of the DS, 29.4% of *Informed* participants did not break away from their familiar strategy. We suggest that prior experience with school-like testing, or other stable problem-solving situations may play a key role in cognitive set bias. Future studies should directly test this.

It is also possible that, on the LS-DS task, cognitive set is driven by a desire to respond “appropriately”, rather than efficiently^51^. In other words, we did not distinguish between participants believing that they *could* not versus *should* not use the shortcut. However, from the questionnaires, it seemed that even participants who reported noticing the difference between BASE and PROBE trials and/or valuing the Triangle, did not consider the shortcut a viable solution until after watching the DS video (Supplementary Table S4). Additionally, none of participants’ responses suggested active avoidance of the shortcut.

We conclude that cognitive set on the LS-DS task is not attributable to an inability to detect the alternative but rather to participants’ understanding of the problem space and their (un)willingness to explore alternatives. We suggest that prior experience with rule-based problem solving, especially in the context of formal education, might lead to increased set. The impact of rote memorization and mechanized rule-use, typical of Western educational approaches, on cognitive inflexibility should be clearly elucidated in future studies.

*“When the individual does not adequately deal with problems but views them merely from the frame of reference of a habit; when he applies a certain habituated behavior to situations which have a better solution or which, in fact, are not even solvable by the just working habit; when, in a word, instead of the individual mastering the habit, the habit masters the individual – then mechanization is indeed a dangerous thing.” (Luchins, 1942, p. 93)*

## Supplementary Information

Supplementary Information.

## Data Availability

Data is publicly available through the Harvard Dataverse here.
