# Supplementary material for: Overcoming cognitive set bias requires more than seeing an alternative strategy
Source: Sci Rep. 2022 Feb 9;12:2179. doi: 10.1038/s41598-022-06237-0 (PMC8828898; doi:10.1038/s41598-022-06237-0)
Supplement: Supplementary file 1 — Supplementary Information. [file 41598_2022_6237_MOESM1_ESM.pdf]

Title: Overcoming cognitive set bias requires more than seeing an alternative strategy
Authors: Sarah M. Pope-Caldwell, David A. Washburn

Supplementary Materials

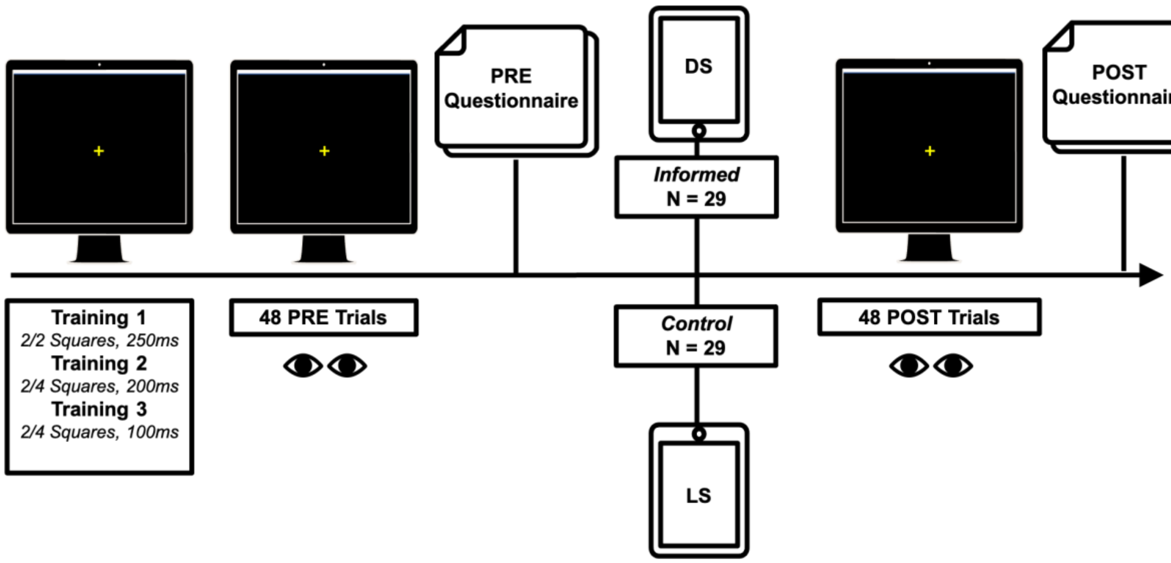

Figure S1: Study procedure. Procedure time-line. After passing Training 1, Training 2, and Training 3, all participants conducted 48 PRE trials, followed by a PRE questionnaire. Next, *Informed* participants watched the DS video and *Control* participants watched the LS video. Finally, all participants conducted 48 POST trials, followed by a POST questionnaire. Gaze was recorded during PRE and POST trial blocks.

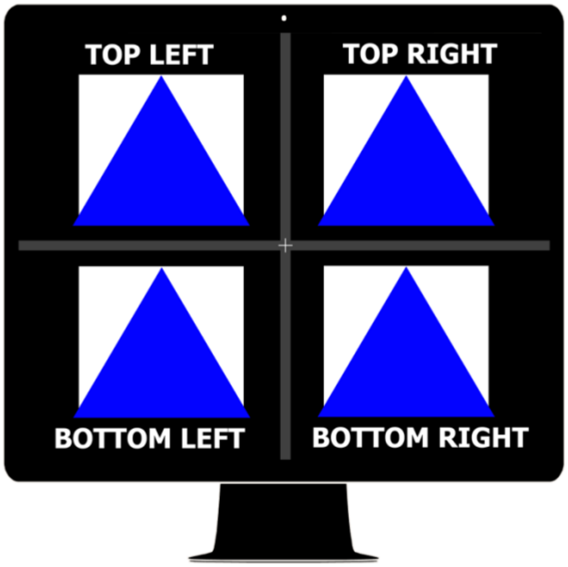

Figure S2: Determining of gaze locations. Response screen divided into quadrants to identify gaze location. The squares and triangles illustrate where stimuli appeared within each quadrant. The middle gray line signifies the 30-pixels-wide line within which fixations were excluded.

Table S1: Models 1.0 & 1.1 investigating the impact of trial type (BASE and PROBE) on Triangle fixations prior to the first response. a) WAIC estimates and standard error comparison. **b)** Model output; estimates with 95% credible intervals that did not include zero are bolded.

a)

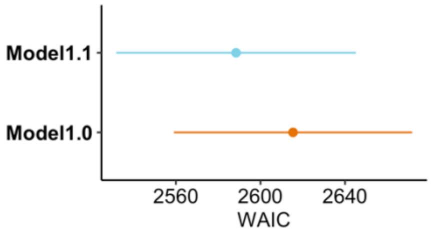

b)

		Est.	Est. Error	l-95% CI	u-95% CI
Model 1.0	**Intercept**	**-1.41**	**0.07**	**-1.55**	**-1.26**
Model 1.1	**Intercept**	**-1.68**	**0.09**	**-1.86**	**-1.51**
	**PROBE vs BASE**	**0.53**	**0.10**	**0.34**	**0.72**

Table S2: Models S1, S2, & S3 investigating the impact of trial type (BASE and PROBE) on Square1, Square2, and Foil fixations, prior to response 1. Estimates with 95% credible intervals that did not include zero are bolded.

		Est.	Est. Error	l-95% CI	u-95% CI
Model S1 (*Square 1*)	Intercept	-0.05	0.10	-0.25	0.15
	**PROBE vs BASE**	**-0.17**	**0.08**	**-0.33**	**-0.01**
Model S2 (*Square 2*)	**Intercept**	**-1.31**	**0.09**	**-1.49**	**-1.12**
	PROBE vs BASE	-0.15	0.10	-0.34	0.04
Model S3 (*Foil*)	**Intercept**	**-1.67**	**0.09**	**-1.85**	**-1.5**
	PROBE vs BASE	-0.01	0.11	-0.22	0.2

Table S3: Models 2.0, 2.1 & 2.2 investigating the impact of receiving the video information on participants' shortcut-use. a) WAIC estimates and standard error comparison. **b)** Model output; estimates with 95% credible intervals that did not include zero are bolded.

a)

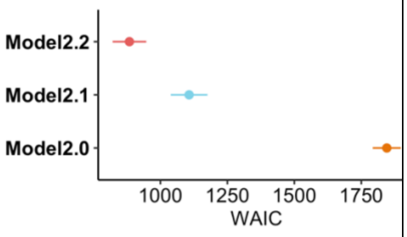

b)

		Est.	Est. Error	l-95% CI	u-95% CI
Model 2.0	**Intercept**	**-1.57**	**0.37**	**-2.27**	**-0.82**
Model 2.1	**Intercept**	**-5.09**	**0.69**	**-6.42**	**-3.68**
	**POST vs PRE**	**3.98**	**0.19**	**3.60**	**4.37**
	**Informed vs Control**	**2.62**	**0.96**	**0.70**	**4.44**
Model 2.2	**Intercept**	**-3.23**	**0.65**	**-4.44**	**-1.90**
	POST vs PRE	0.20	0.27	-0.33	0.74
	Informed vs Control	-0.08	0.96	-2.01	1.81
	**POST Informed : PRE Control**	**6.06**	**0.44**	**5.23**	**6.93**

Table S4: Participants self-reports of noticing and valuing the shortcut. Point-biserial correlations between shortcut use and **a)** participants' reports of noticing the Triangle in PRE trials, **b)** participants' reports of valuing the Triangle in PRE trials, **c)** participants' reports of noticing the Triangle in POST trials, **d)** participants' reports of valuing the Triangle in POST trials.

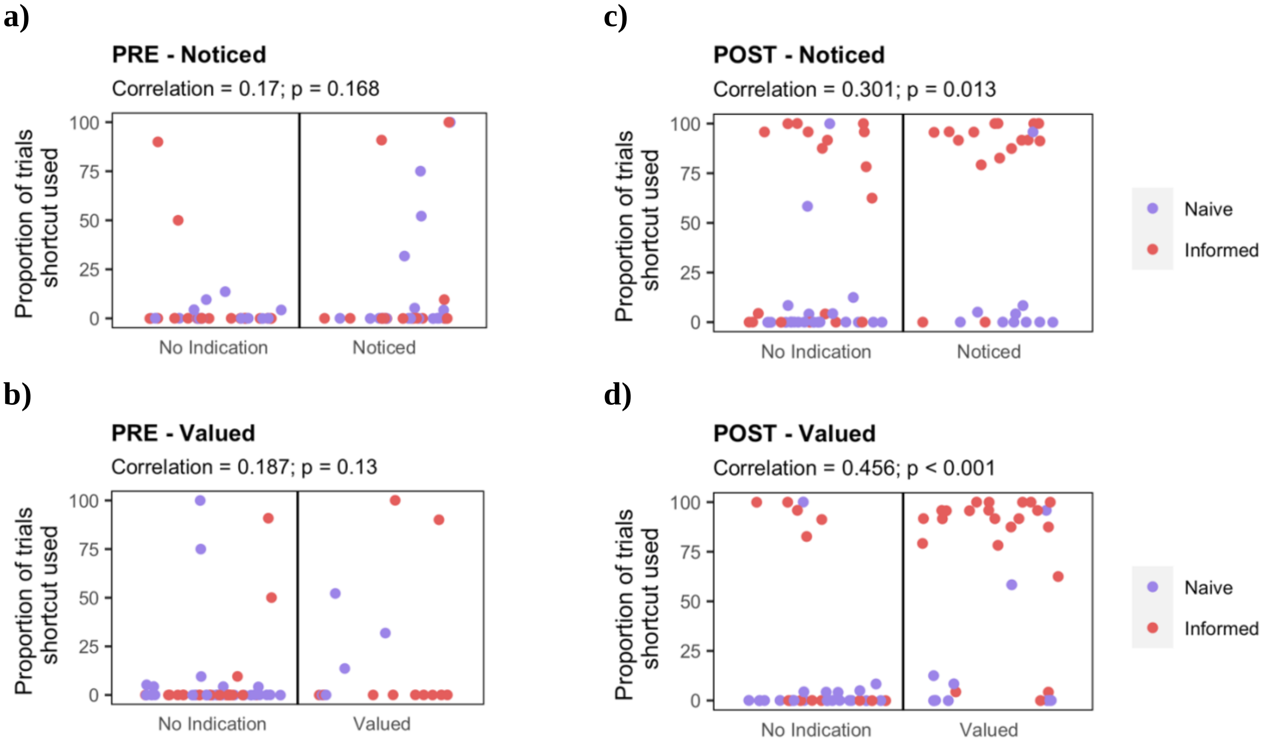

Table S5: Models 3.0 & 3.1 investigating the impact of strategy on trial duration. a) WAIC estimates and standard error comparison. b) Model output; estimates with 95% credible intervals that did not include zero are bolded.

a)

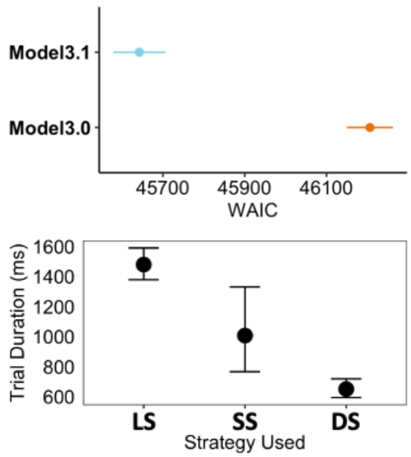

b)

		Est.	Est. Error	l-95% CI	u-95% CI
Model 3.0	Intercept	7.15	0.04	7.07	7.23
Model 3.1	**Intercept**	**7.30**	**0.04**	**7.23**	**7.37**
	**LS vs SS**	**-0.38**	**0.14**	**-0.65**	**-0.11**
	**LS vs DS**	**-0.82**	**0.04**	**-0.90**	**-0.74**

Table S6: Models 4.0, 4.1 & 4.2 investigating the effects of switching between PROBE DS and BASE LS trials, on participants' latency to first response. a) WAIC estimates and standard error comparison. **b)** Model output; estimates with 95% credible intervals that did not include zero are bolded.

a)

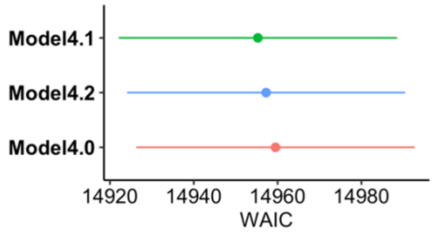

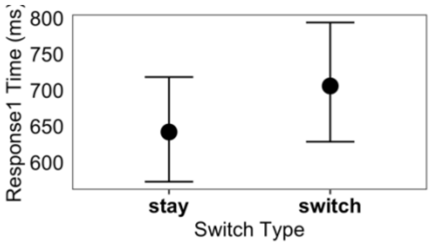

b)

		Est.	Est. Error	l-95% CI	u-95% CI
Model 4.0	**Intercept**	**6.49**	**0.05**	**6.40**	**6.58**
Model 4.1	**Intercept**	**6.47**	**0.06**	**6.35**	**6.58**
	Stay vs Switch	0.09	0.05	-0.01	0.20
	BASE vs PROBE	-0.05	0.05	-0.16	0.05
Model 4.2	**Intercept**	**6.45**	**0.06**	**6.33**	**6.57**
	Stay vs Switch	0.12	0.07	-0.02	0.26
	BASE vs PROBE	-0.02	0.07	-0.17	0.13
	Stay BASE : Switch PROBE	-0.06	0.10	-0.26	0.15

Table S7. The number of participants that used the DS or SS shortcuts in the first PROBE trial (First), more than 5% of PROBE trials (5% or more), more than 50% of PROBE trials (50% or more), and every correct trial (100%) for PRE and POST blocks.

		**First**	**5% or more**	**50% or more**	**100%**	**Total**
**PRE**	Naive	1	7	3	1	33
	Informed	2	5	4	1	34
**POST**	Naive	2	7	3	1	33
	Informed	17	24	24	7	34
